# 1-Deoxynojirimycin (DNJ) Ameliorates Indomethacin-Induced Gastric Ulcer in Mice by Affecting NF-kappaB Signaling Pathway

**DOI:** 10.3389/fphar.2018.00372

**Published:** 2018-04-19

**Authors:** Xuehua Piao, Shuangdi Li, Xiaodan Sui, Lianyi Guo, Xingmei Liu, Hongmei Li, Leming Gao, Shusheng Cai, Yanrong Li, Tingting Wang, Baohai Liu

**Affiliations:** ^1^Department of Traditional Chinese Medicine, The First Affiliated Hospital, Jinzhou Medical University, Jinzhou, China; ^2^Heart Disease Center, The Affiliated Hospital of Changchun University of Traditional Chinese Medicine, Changchun, China; ^3^Department of Hepatology, The Affiliated Hospital of Changchun University of Traditional Chinese Medicine, Changchun, China; ^4^Department of Gastroenterology, The First Affiliated Hospital of Jinzhou Medical University, Jinzhou, China; ^5^School of Stomatology, 2nd Dental Center, Peking University, Beijing, China

**Keywords:** NF-κB signaling pathway, mouse, gastric ulceration, DNJ, prostaglandin E2

## Abstract

Gastric ulcer (GU) is a main threat to public health. 1-Deoxynojirimycin (DNJ) has antioxidant and anti-inflammatory properties and may prevent GU but related mechanism remains unclear. DNJ was extracted from the supernatants of *Bacillus subtilis* by using ethanol and purified by using CM-Sepharose chromatography. A GU mouse model was induced by indomethacin. The functional role of DNJ in GU mice was explored by measuring the main molecules in the NF-KappaB pathway. After the model establishment, 40 GU mice were evenly assigned into five categories: IG (received vehicle control), LG (10 μg DNJ daily), MG (20 μg DNJ daily), HG (40 μg DNJ daily), and RG (0.5 mg ranitidine daily). Meanwhile, eight healthy mice were assigned as a control group (CG). After 1-month therapy, weight and gastric volume were investigated. The levels of serum inflammatory cytokines (IL-6 and TNF-α), antioxidant indices [superoxide dismutase (SOD), catalase (CAT), and reduced glutathione (GSH)], and oxidant biomarker malondialdehyde (MDA) were examined via ELISA. Meanwhile, inflammatory cytokine (IL-6 and TNF-α) levels, and key molecules (NF-κB p65), cyclooxygenase 1 (COX-1 and COX2) involved in NF-κB pathway, were analyzed by using Western Blot. COX-1 and COX-2 levels were further measured by immunohistochemistry. The effects of DNJ on gastric functions were explored by measuring the changes of Motilin (MOT), Substance P (SP), Somatostatin (SS), and Vasoactive intestinal peptide (VIP) in GU mouse models with ELISA Kits. The results indicated that DNJ prevented indomethacin-caused increase of gastric volume. DNJ improved histopathology of GU mice when compared with the mice from IG group (*P* < 0.05). DNJ consumption decreased the levels of IL-6 and TNF-α (*P* < 0.05). DNJ increased antioxidant indices of GU mice by improving the activities of SOD, CAT and reduced GSH, and reduced MDA levels (*P* < 0.05). DNJ increased the levels of prostaglandin E2, COX-1, COX2, and reduced the levels of and NF-κB p65 (*P* < 0.05). DNJ showed protection for gastric functions of GU mice by reducing the levels of MOT and SP, and increasing the levels of SS and VIP. DNJ treatment inactivates NF-κB signaling pathway, and increases anti-ulceration ability of the models.

## Introduction

Gastric ulcer (GU) is a digestive disease in stomach lining and its common symptoms are burning, dull ache, and waking at night. GU is also associated with vomiting (Radovanovic et al., 2014), abdominal pain, fever, poor oral tolerance, and weight loss (Espinoza-Rios et al., 2017). Gastric bleeding occurs in many GU patients and is often hard to be treated (Barola et al., 2017; Godina et al., 2017; Pang and Hagen, 2017; Xing et al., 2017). GU has become a main threat to public health and greatly affects the life quality of patients. However, pathological manifestation of GU is often varied and the exact pathogenesis of GU has not been fully elucidated.

Medicine therapy is still the main choice in GU treatment of but most medicine has remarkable side effects, which inhibit its clinical use. Gastric acid inhibition is often used for preventing GU bleeding. Somatostatin and pantoprazole are effective for inhibiting gastric acid secretion. Comparatively, somatostatin is more effective than pantoprazole in maintaining high intragastric pH (Avgerinos et al., 2005). However, long-term use of pantoprazole can result in thrombocytopenia (Tas, 2013). Somatostatin therapy often causes hypoglycemia in a sudden and unexpected way (Navascues et al., 1988). Sucralfate is a drug to treat GU and reduces leukocyte adherence in post-capillary venule, TNF-α level and inflammation, and promotes GU healing in an animal model (Eamlamnam et al., 2006), whereas the drug often causes nausea, vomiting, diarrhea, dizziness, insomnia, and headache (Dugan, 1991). Omeprazole belongs to proton pump inhibitors and is proved effective for treating GU syndrome (Bush et al., 2018). Early work found that hemifacial paralysis might be an adverse effect associated with omeprazole treatment in a lymphoblastic leukemia patient (Bauters et al., 2010). Furthermore, severe adverse effects, including an allergic shock, rash and diarrhea, could be caused by the accumulation of omeprazole metabolites (Yu et al., 2016).

*Helicobacter pylori* infection and the long-term use of non-steroidal anti-inflammatory medicine often contribute to GU progression. Antibiotics are often considered for GU therapy. However, drug resistance and the side effects have become a global problem and it is necessary to find a new method to treat GU (Khoder et al., 2016). Meanwhile, exploring alternative antibiotics with few side effects has become very urgent.

According to earlier reports, oxidative stress plays an important role in the pathogenesis of GU (Chen et al., 2016) because it can cause oxidative damage in gastric tissue (Boyacioglu et al., 2016). DNJ is an effective α-glucosidase inhibitor and has been reported that administration of DNJ suppresses an increase in post-prandial blood glucose in humans. Intake of DNJ reduces lipid level and leads to a reduction of oxidative stress (Tsuduki et al., 2009). CAT, reduced GSH, and SOD are important antioxidant biomarkers whereas MDA is an important oxidative biomarker. Thus, DNJ may affect the levels of the oxidation-related molecules. DNJ can be isolated from mulberry (Jiang et al., 2014; Wang et al., 2014, 2015; E et al., 2017; Hu et al., 2017) or produced by *Bacillus* species (Lee et al., 2013; Seo et al., 2013; Do et al., 2015; Cai et al., 2017). DNJ may be a potential drug for treating post-prandial hyperglycemia with few side effects (Asai et al., 2011; Jiang et al., 2014). With the study on DNJ, it has been found to have many health promoting properties. DNJ derivative can inhibit dengue virus infection, which was approved both *in vitro* and *in vivo* (Yu et al., 2012). Further work indicates that DNJ has an inhibitory effect against virulence pathways of bacteria (Hasan et al., 2014) and anti-pathogen activities (Shaheen et al., 2006; Gunjal et al., 2015). Its derivative the glycolipid biosynthesis inhibitor was found to be with strong anti-inflammatory and immune suppressive properties on both trinitrobenzene sulfonic acid – and oxazolone (4-ethoxymethylene-2phenyl-2oxazoline-5-one; Oxa)-induced colitis (Shen et al., 2004). DNJ also can reduce the levels of tumor necrosis factor α (TNF-α), interleukin-1 (IL-1), interleukin-6 (IL-6) in liver organ (Liu et al., 2016). However, the effects of DNJ on GU and related molecular mechanism remain unclear.

Nuclear factor-κB (NF-κB) p65, an important transcription factor, is involved with many immune and inflammatory activities. NF-κB p65 is a pivotal transcription factor of M1 macrophages and induces a great amount of inflammatory genes, including TNF-α, IL-1β, IL-6, IL-12, and cyclooxygenase-2 (Jeoung et al., 2013; Jeong et al., 2017). Deregulation of MAPK/NF-κB signal pathway-related proteins p-ERK, p-JNK, p-p38, p-IκB, and p-NF-κB p65 is supposed to have protective functions for controlling GU development (Chang et al., 2015; Akanda and Park, 2017). Previous studies indicated NF-κB signaling is associated with pathogenesis and progression of GU (Mahmoud-Awny et al., 2015). Prostaglandin E2 (PEG2) can affect the activity of NF-κB pathway (Dilshara et al., 2016; Morikawa et al., 2016; Aoki et al., 2017). Therefore, inactivation of NF-κB signaling pathway via PEG2 may have beneficial effects for GU treatment. Therefore, the effects of DNJ on GU were explored by exploring oxidative parameters and the main molecules in NF-κB signaling pathway.

## Materials and Methods

### Purification of DNJ

*Bacillus subtilis* was isolated from soybean and identified by using 16S rDNA sequence analysis. One colony of the bacteria culture was cultured in 1 L LB medium at 37°C and 200 rpm shaking for 96 h. The supernatant was collected after centrifugation at 15,000 *g* for 10 min. Ethanol was added to final concentration up to 80%. In order to precipitate the polysaccharide effectively, the solution was set at 4°C for 1 h. After centrifugation at 15,000 *g* for 10 min, the supernatant was concentrated to 50 mL, and dried by using vacuum evaporation and then immediately freeze-dried. The lyophilized powder was dissolved in 50 mL of distilled water and filtered with a 0.22-μm membrane (Millipore, Corp., Bedford, MA, United States). The solution was further ultra-filtrated by using 1 kDa molecular weight cutoffs (MWCO) (Millipore). Active charcoal (Sigma, St. Louis, MO, United States) was placed in a column (2.5 cm × 40 cm) and the column was equilibrated with ddH_2_O for 2 h at a flow rate of 2 mL/min. The sample solution was loaded onto the column and 0-50% of ethanol was used for the gradient elution at a rate of 1 mL/min. Eluted solution was collected at 2-min intervals, and individual fraction confirmed by measuring α-glucosidase inhibitory activities. Targeted fraction was further purified by using CM-Sepharose chromatography (5 cm × 20 cm, Sigma). DNJ was dissolved in formic acid solution (pH 4) and loaded onto the column. Finally, DNJ was eluted by using formic acid with various pH and confirmed by measuring α-glucosidase inhibitory activity according to an earlier report (Yatsunami et al., 2008). The eluted DNJ was dried by using a vacuum freeze-dryer (Tofflon, Shanghai, China). DNJ purity was analyzed by using a high-performance liquid chromatography (HPLC). Purified DNJ was dissolved in acetonitrile and water (1:1, v/v) with 20 mM NH_4_HCO_3_. Ten μL solution was injected into HPLC (Jasco, Tokyo, Japan) with a unison United Kingdom-amino column (2.0 mm × 100 mm, 3-μm particle diameter, Imtakt Corporation, Kyoto, Japan). A mobile phase [acetonitrile and water (9:1, v/v) with 20 mM NH_4_HCO_3_] was used and ran through at a flow rate of 1 mL/min.

### Animals

All processes were approved by the Animal Research Ethical Committee of Jinzhou Medical University (Approval No. 20160609y, Jinzhou, China). A total of 48 male mice (C57BL/6J, 5 weeks) weighing 18–20 g were purchased from Experimental Animal Center of Jinzhou Medical University Mice and kept under an automated 12-h light–dark cycle at a controlled temperature of 22 ± 2°C, and relative humidity of 50–60%. All mice had *ad libitum* access to a standard dry diet and tap water.

### GU Induction and Animal Grouping

A GU mouse model was established according to a previous report (Chatterjee et al., 2013). The mice had no food but free access to water 24 h before GU induction. A total of 40 mice received 18 mg/kg indomethacin (dissolved in distilled water and suspended in 2% gum acacia) for 5 consecutive days, and eight mice received 2% gum acacia as a control group (CG). After the establishment of GU model, eight model mice (received saline solution) were assigned as an indomethacin-induced control group (IG). Twenty-four mouse models received different concentrations of DNJ (10, 20, and 40 μg/kg dissolved in saline solution once daily) were assigned as LG, MG, and HG groups. Eight mouse models were administrated with ranitidine (0.5 mg daily) as a positive control group (RG). The mouse from CG group received saline solution. The diets were given among these groups as **Table 1** showed and there was no difference for other components except of DNJ and ranitidine. After 1-month treatment, the mice were killed by using cervical dislocation following anesthesia (sodium thiopental, 60 mg/kg). The stomach was isolated and opened along the greater curvature, and rinsed with 0.85% saline solution.

**Table 1:** Composition of experimental diets Ingredient (g/kg).

Ingredients	CG	Gastric ulceration models				
		IG	LG	MG	HG	RG
Atlantic salmon	200	200	200	200	200	200
Casein	35	35	35	35	35	35
L-Cystine	33	33	33	33	33	33
Corn starch	386.5	386.5	386.5	386.5	386.5	386.5
Maltodextrin	132	132	132	132	132	132
Sucrose	100	100	100	100	100	100
Fruit and vegetable powder	60	60	60	60	60	60
Soybean oil	6	6	6	6	6	6
Cellulose	30	30	30	30	30	30
Mineral mix, AIN-93G-MX	35	35	35	35	35	35
Vitamin mix, AIN-93-VX	10	10	10	10	10	10
Choline bitartrate 2.5	2.5	2.5	2.5	2.5	2.5	2.5
*tert*-Butylhydroquinone	0.014	0.014	0.014	0.014	0.014	0.014
DNJ (μg)	–	–	10	20	40	–
Ranitidine (mg)	–	–	–	–	–	0.5
Water	238	238	238	238	238	238

*After the establishment of gastric ulcer model, eight model mice (received saline solution) were assigned as an indomethacin-induced control group (IG). Other model mice received different concentrations of DNJ (10, 20, and 40 μg/kg dissolved in saline solution once daily) were assigned as LG, MG, and HG groups. The model mice were administrated with ranitidine (0.5 mg daily) as a positive control group (RG). Healthy mice without indomethacin induction were regarded as the CG group. n = 8 in each group.*

### Determination of Stomach Capacity

Gastric volume of the mouse was measured by filling the stomach with distilled water, and a crude assessment of volume was made. To make sure the exact result, no stress on the stomach, or stretched, and or bursting.

### GU Index

The damage score of GU were measured by using gastric injury on a 0–4 scale (Erkin et al., 2006) according to the severity of hyperemia and hemorrhagic erosion: 0 = normal mucosa; 0.5 = hyperemia; 1 = one or two lesions; 2 = severe lesions; 3 = very severe lesions; 4 = full of lesions. The data were presented as mean values ± standard deviation (SD).

### Measurement of GU

Gastric ulcer degree was measured by according to an earlier report (Sabiu et al., 2015). Briefly, cleaned stomachs were pinned on a cork board and GC degrees were observed under a dissecting microscope on a 0–5 scale given in the reference (Sabiu et al., 2015). Damage areas of gastric mucosa were showed as the percentage of the total area of the glandular stomach. Ulcer index (U.I) was presented as a mean ulcer score and the percentage of inhibition against ulceration was calculated by using the equation: U.I. = (Ulcerated area/total stomach area) × 100%. Ulcer inhibition = (U.I. in CG – U.I. in mouse models) 100/U.I. in CG.

### Measurement of Antioxidant Indices

After ulcer scoring, one cm segment from the distal stomach was taken for subsequent histological analysis. Whole stomach tissue was ground with liquid nitrogen using a mortar and a pestle. The ground tissue (500 mg each) was homogenized in 100 mM PBS (pH 7.0) and the homogenate was centrifuged at 8,000 rpm for 20 min at 4°C. The activity of superoxide dismutase (SOD) was examined by using SOD Assay Kit (Cayman Chemicals, Ann Arbor, MI, United States). Reduced Glutathione (GSH) (Category No. ab65322), Catalase (CAT) (Category No. ab118184), and Malondialdehyde (MDA) (Category No. ab118970) levels were detected by using the kits from Abcam’s Shanghai branch (Shanghai, China).

### Histology Analysis of Gastric Damage

For histological examination, 1 cm segment from the distal stomach was fixed in 4% formalin, embedded in paraffin blocks and cut into 4 μm-thick sections and placed on glass slides. The sections were stained with hematoxylin and eosin (H&E) to study histological changes. Background stain could be blurry and H&E staining was necessary for to visualize histopathological features of GU issues. Stained stomach section was observed under a light microscope (XSP148AT; Shanghai Taiyi Medical Apparatus Equipment, Co., Ltd., Shanghai, China).

### Measurement of Inflammatory Cytokines

One-half milliliter of blood was obtained from mouse tail vein before it was killed and serum was isolated by centrifuged at 2500 rpm for 10 min. The concentrations of IL-6 (Category No. ab100713) and TNF-α (Category No. ab46105) were measured by using ELISA kits from Abcam.

### Analysis for the Effects of DNJ on Gastric Functions

Motilin (MOT) modulates gastric contraction via cholinergic, adrenergic, serotonergic, and NO neurons (Mondal et al., 2011), and is associated with emptying the stomach during the interdigestive period (Dudani et al., 2016). Substance P (SP) belongs to an undecapeptide member of the tachykinin neuropeptide family. The total quantity of SP of stomach can be measured in the mucous membrane, especially in pylorus the muscular coat with the largest amount of SP (Pernow, 1951). Somatostatin (SS) is growth hormone–inhibiting hormone and a peptide hormone regulating the endocrine system, and affecting neurotransmission by interacting with G protein-coupled SS receptors and inhibiting hormone release (Yamasaki et al., 2017). Somatostatin inhibits insulin and glucagon secretion. SS changes occur in the neurons supplying the prepyloric area of the stomach during adaptation to various pathological processes (Palus et al., 2017). Vasoactive intestinal peptide regulates digestion process, control blood pressure and heart rate, and shows cardio- and gastro-protective character (Kasacka et al., 2015). From the information, these proteins are all associated with stomach functions. To understand the effects of DNJ on gastric functions, the effects of DNJ on MOT, SP, SS, and VIP in GU mouse models were measured by using Mouse Motilin ELISA Kit from XpressBio (Category No. SKU: XPEM1219, Frederick, MD, United States), Mouse SS (Somatostatin) ELISA Kit from XpressBio (Category No. SKU: XPEM0417), Mouse Substance P ELISA Kit from RayBiotech (Category No. EIAM-SP-1, Norcross, GA, United States), and Human, Mouse, Rat VIP ELISA Kit from RayBiotech (Category No. EIA-VIP-1).

### Determination of PGE 2

For PGE 2 level in the homogenate of stomach tissue, the supernatant was measured by using an ELISA kit from Cayman Chemical (Ann Arbor, MI, United States) after centrifugation at 12000 *g* for 10 min. The optical densities were measured at a wavelength of 450 nm and the results were expressed as ng/g protein.

### Immunohistochemistry for COX-1 and COX-2

Slides of paraffin-embedded gastric tissues were deparaffinized in xylene and rehydrated in a graded ethanol series and then under running water for 5 min. The inhibition of endogenous peroxidase activity was followed by antigen retrieval using microwave heating. Slides were incubated with specific primary antibodies that recognized either COX-1 (dilutions 1:200) or COX-2 antibody (dilutions 1:500). Overnight, sections were then incubated with biotinylated anti-mouse secondary antibody followed by peroxidase-labeled streptavidin-horse radish protein (HRP) complex and 3,3,0-diaminobenzidine tetra hydrochloride (DAB). COX-1 (Category No. ab2388), COX-2 (Category No. ab15191), and IgG H&L (horseradish peroxidase, Category No. ab97051 from Abcam) were purchased from Abcam (Cambridge, MA, United States). The specimens were counterstained with H&E staining, and examined under a light microscope. The mean value of integrated optical density (IOD) was calculated using Image-Pro Plus software (IPP, version 6.0, Media Cybernetics, Inc.).

### Western Blot Analysis

Proteins were extracted from gastric homogenate by using protein extraction kits (Beyotime, Beijing, China). All proteins were separated on SDS-PAGE (12% gel), and transferred to a PVDF membrane (Millipore, Corp., Bedford, MA, United States) and blocked in 5% milk for 20 min. The membrane was incubated with rabbit-derived first antibodies for NF-κB p65 (Category No. ab16636), TNF-α (Category No. ab9635), COX-1 (Category No. ab2388), COX-2 (Category No. ab15191), IL-6 (Category No. ab9324), and GAPDH (Category No. ab9485) from Abcam (Cambridge, MA, United States). The blot was labeled with Goat Anti-Rabbit IgG H&L (horseradish peroxidase, Category No. ab97051 from Abcam). Signals were quantified by ImageJ 1.40 (NIH, Bethesda, MA, United States).

### Statistical Analysis

All data were shown as mean values ± standard derivative (S.DNJ.). Statistical analysis was carried out by using SPSS 20.0 soft package from IBM Corporation (Armonk, NY, United States). *t*-Test was used to compare the significance of the variables between two groups. The statistical differences are significant if *P* < 0.05.

## Results

### DNJ Purity

**FIGURE 1:**
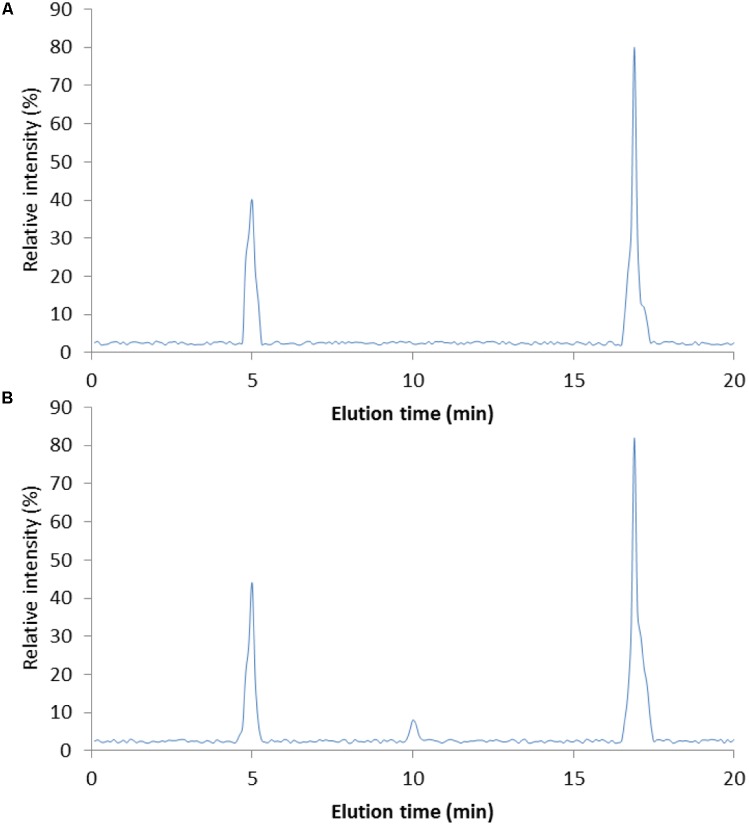
High-performance liquid chromatography (HPLC) analysis of DNJ purity. **(A)** DNJ standard. **(B)** DNJ was purified from *Bacillus subtilis*. HPLC, high-performance liquid chromatography; DNJ, 1-deoxynojirimycin. DNJ was eluted at 16.9 min.

**Figure 1** showed the purified DNJ (**Figure 1B**) when compared with DNJ standard (**Figure 1A**). On the chromatogram, the solvent peak appeared at about 5 min and the retention time of the main peak was 16.9 min, which was similar with that of DNJ standard.

### DNJ Controlled the Increase of Gastric Volume Induced by Indomethacin

**FIGURE 2:**
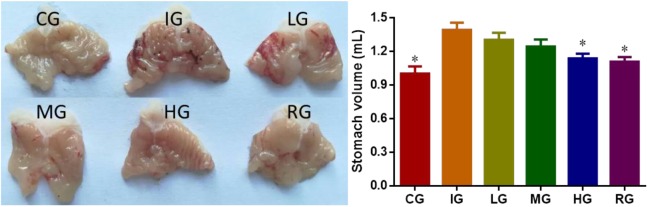
The effects of DNJ on stomach volume of GU mouse models. Eight healthy mice were assigned as a control group (CG) and eight model mice were assigned as a model group (IG). The model mice received different concentrations of DNJ (10, 20, and 40 μg/kg daily) were assigned as LG, MG, and HG groups. The model mice were administrated with ranitidine (0.5 mg daily) as a positive control group (RG). *N* = 8 for each group. ^∗^*P* < 0.05 vs. the IG group.

Indomethacin administration caused significant increase in gastric volume (**Figure 2**, *P* < 0.05). To explore the effects of DNJ on the gastric volume of GU mouse models, a series of concentrations (0, 10, 20, and 40 μg daily, respectively) of DNJ were used. The results showed that 40-μg DNJ pre-treatment caused significant decrease in gastric volume when compared with other GU models (**Figure 2**, *P* < 0.05). The results suggest DNJ controls the increase of gastric volume induced by indomethacin with the increase in the dosage of DNJ.

### DNJ Reduced Ulceration Index in GU Mice

**FIGURE 3:**
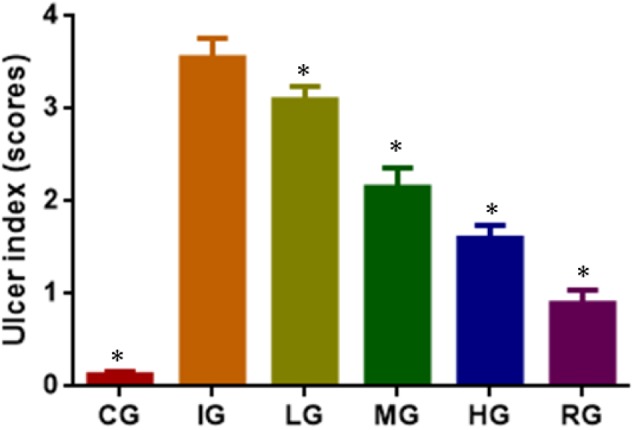
The effects of DNJ on ulceration index in GU mouse model. Eight healthy mice were assigned as a CG and eight model mice were assigned as a model group (IG). The model mice received different concentrations of DNJ (10, 20, and 40 μg/kg daily) were assigned as LG, MG, and HG groups. The model mice were administrated with ranitidine (0.5 mg daily) as a positive control group (RG). *N* = 8 for each group. ^∗^*P* < 0.05 vs. the IG group.

Indomethacin administration increased ulceration score of GU models (**Figure 3**, *P* < 0.05). The increase could be controlled by using DNJ and the scores were reduced with the increase in DNJ dosage. The statistical differences for ulceration index were significant between the IG group and CG or HG, and or RG groups (**Figure 3**, *P* < 0.05).

### DNJ Reduced Ulceration Development in GU Mice

**FIGURE 4:**
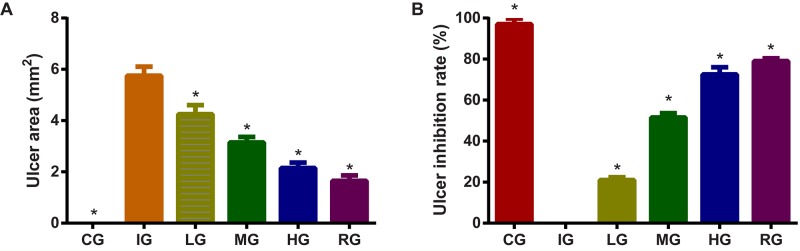
The effects of DNJ on ulceration area and rate in GU mouse model. **(A)** The effects of DNJ on ulceration area in GU mouse model. **(B)** The effects of DNJ on ulceration rate in GU mouse model. Eight healthy mice were assigned as a CG and eight model mice were assigned as a model group (IG). The model mice received different concentrations of DNJ (10, 20, and 40 μg/kg daily) were assigned as LG, MG, and HG groups. The model mice were administrated with ranitidine (0.5 mg daily) as a positive control group (RG). *N* = 8 for each group. ^∗^*P* < 0.05 vs. the IG group.

Indomethacin administration increased ulceration area (**Figure 4A**, *P* < 0.05) and ulceration rate (**Figure 4B**, *P* < 0.05) of GU mouse models. GU was established by increasing ulcer area after indomethacin induction. The increase could be controlled by using DNJ and GU development was controlled with the increase in DNJ dosage. The statistical differences for ulceration area and rates were significant between the IG group and CG or HG, and or RG groups (**Figures 4A,B**, *P* < 0.05). DNJ shows protection against ulceration by reducing ulcer area and strong ulcer inhibitory rates (**Figure 4**, *P* < 0.05) and anti-ulceration properties.

### DNJ Protected Against the Stomach Damage Caused by Indomethacin

**FIGURE 5:**
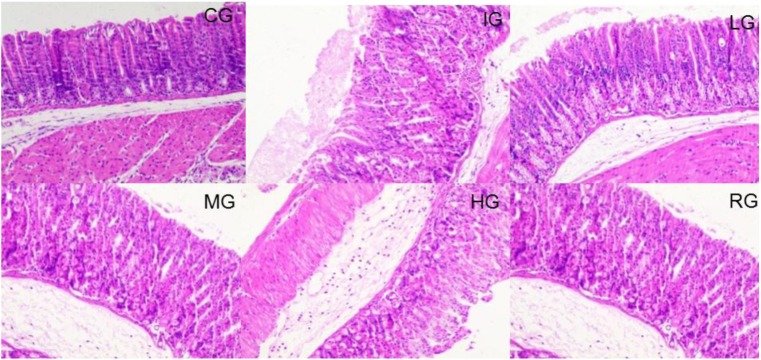
H&E staining analysis of the effects of DNJ on the stomach damage caused by indomethacin in GU mouse model. Eight healthy mice were assigned as a CG and eight model mice were assigned as a model group (IG). The model mice received different concentrations of DNJ (10, 20, and 40 μg/kg daily) were assigned as LG, MG, and HG groups. The model mice were administrated with ranitidine (0.5 mg daily) as a positive control group (RG). *N* = 8 for each group.

H&E analysis showed that tissue structure was in normal form with perfect cell nucleus and cell plasm (**Figure 5**). Indomethacin treatment caused tissue damage with destroyed cells whereas DNJ reduced the damage severity with the increase in its dosage. For histological scores, the statistical differences were significant between IG and CG, or HG and or RG groups (**Figure 5**, *P* < 0.05).

### DNJ Inhibited Serum Levels of Inflammatory Factors

**FIGURE 6:**
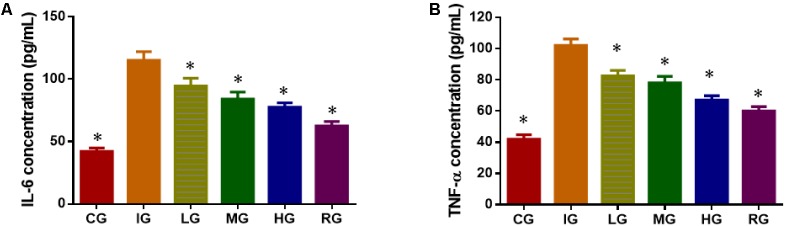
ELISA analysis of the effects of DNJ on serum levels of inflammatory factors in GU mouse model. **(A)** The effects of DNJ on serum levels of IL-6. **(B)** The effects of DNJ on serum levels of TNF-α. Eight healthy mice were assigned as a CG and eight model mice were assigned as a model group (IG). The model mice received different concentrations of DNJ (10, 20, and 40 μg/kg daily) were assigned as LG, MG, and HG groups. The model mice were administrated with ranitidine (0.5 mg daily) as a positive control group (RG). *N* = 8 for each group. ^∗^*P* < 0.05 vs. the IG group.

ELISA analysis showed that indomethacin treatment increased the serum levels of inflammatory factors IL-6 (**Figure 6A**) and TNF-α (**Figure 6B**) when compared with healthy animals (*P* < 0.05). DNJ inhibited the serum levels of inflammatory factors IL-6 and TNF-α with the increase in its dosage, and the statistical differences were significant between IG and other groups (**Figure 6**, *P* < 0.05).

### DNJ Improved Antioxidant Ability of GU Models

**FIGURE 7:**
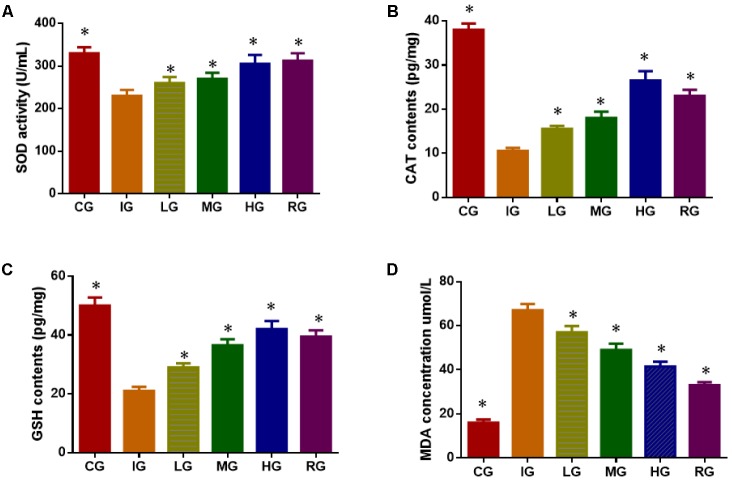
The effects of DNJ on antioxidant indices in GU mouse models. **(A)** The effects of DNJ on the activity of SOD in GU mouse model; **(B)** The effects of DNJ on the activity of CAT in GU mouse model; **(C)** the effects of DNJ on the activity of reduced GSH in GU mouse model; **(D)** the effects of DNJ on the level of MDA in GU mouse model. Eight healthy mice were assigned as a CG and eight model mice were assigned as a model group (IG). The model mice received different concentrations of DNJ (10, 20, and 40 μg/kg daily) were assigned as LG, MG, and HG groups. The model mice were administrated with ranitidine (0.5 mg daily) as a positive control group (RG). *N* = 8 for each group. ^∗^*P* < 0.05 via a CG.

Indomethacin treatment increased oxidative stress of GU models by reducing the levels SOD (**Figure 7A**), CAT (**Figure 7B**), and reduced GSH (**Figure 7C**), increasing the levels of MDA (**Figure 7D**) when compared with healthy animals (*P* < 0.05). Comparatively, DNJ improved antioxidant capacities of GU mice by increasing the levels SOD (**Figure 7A**), CAT (**Figure 7B**), and reduced GSH (**Figure 7C**), and reducing the levels of MDA (**Figure 7D**) with the increase of its dosage (*P* < 0.05). The results suggest DNJ improves antioxidant indices of GU models.

**FIGURE 8:**
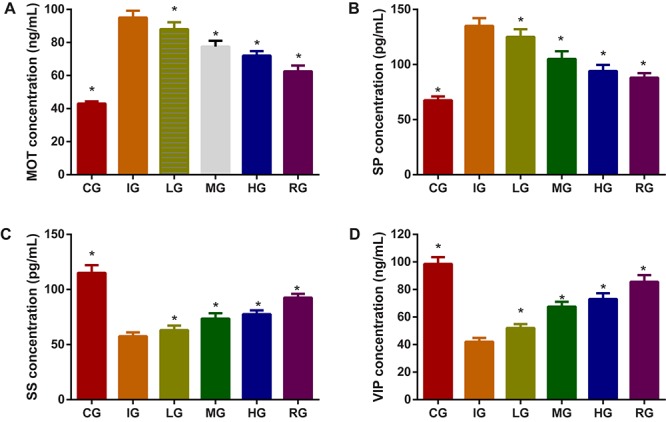
The effects of DNJ on motilin (MOT), substance P (SP), somatostatin (SS), and vasoactive intestinal peptide (VIP) in GU mouse model. **(A)** The effects of DNJ on MOT in GU mouse model. **(B)** The effects of DNJ on substance SP in GU mouse model. **(C)** The effects of DNJ on SS in GU mouse model. **(D)** The effects of DNJ on VIP in GU mouse model. Eight healthy mice were assigned as a CG and eight model mice were assigned as a model group (IG). The model mice received different concentrations of DNJ (10, 20, and 40 μg/kg daily) were assigned as LG, MG, and HG groups. The model mice were administrated with ranitidine (0.5 mg daily) as a positive control group (RG). *N* = 8 for each group. ^∗^*P* < 0.05 via the IG group.

The effects of DNJ on stomach functions of SP, SS and VIP in GU mouse model. Indomethacin treatment increased the levels of MOT (**Figure 8A**) and SP (**Figure 8B**), and reduced the levels of SS (**Figure 8C**) and VIP (**Figure 8D**, *P* < 0.05). Comparatively, DNJ showed protection for stomach functions of GU mice by reducing the levels of MOT (**Figure 8A**) and SP (**Figure 8B**), and increasing the levels of SS (**Figure 8C**) and VIP (**Figure 8D**, *P* < 0.05) with the increase of its dosage.

### DNJ Increased COX Expression

**FIGURE 9:**
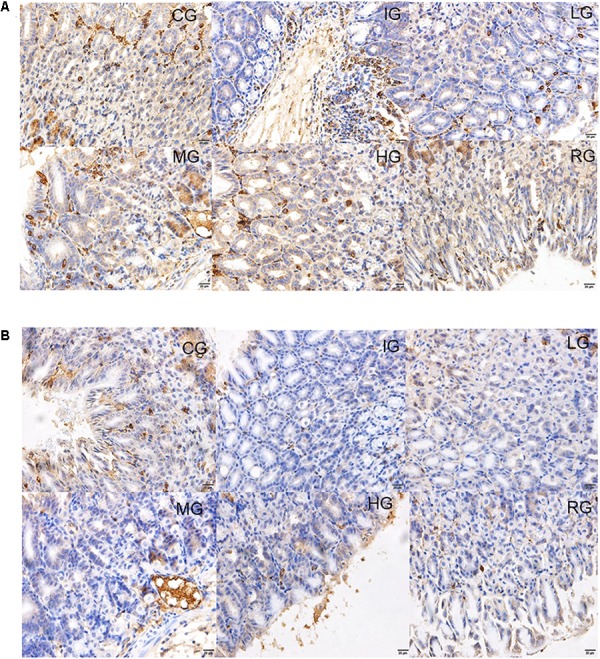
Immunohistological analysis for the effects of DNJ on the expression of COX1 and COX2. **(A)** Immunohistological analysis for the effects of DNJ on the expression of COX1. **(B)** Immunohistological analysis for the effects of DNJ on the expression of COX2. Eight healthy mice were assigned as a CG and eight model mice were assigned as a model group (IG). The model mice received different concentrations of DNJ (10, 20, and 40 μg/kg daily) were assigned as LG, MG, and HG groups. The model mice were administrated with ranitidine (0.5 mg daily) as a positive control group (RG). *N* = 8 for each group.

Immunohistology analysis showed that expression levels of both COX-1 and 2 remarkably reduced by 5.8- and 3.6-fold in mouse models when compared with those in the CG group (**Figures 9A,B**) (*P* < 0.05). Both COX-1 and COX-2 expression were remarkably improved by DNJ in the mucosa on the fourth day after GU induction (**Figures 9A,B**) (*P* < 0.05). The results suggest DNJ treatment improves the expression of COX1 and COX2.

### Effects of DNJ Treatment on PGE2 Synthesis

**FIGURE 10:**
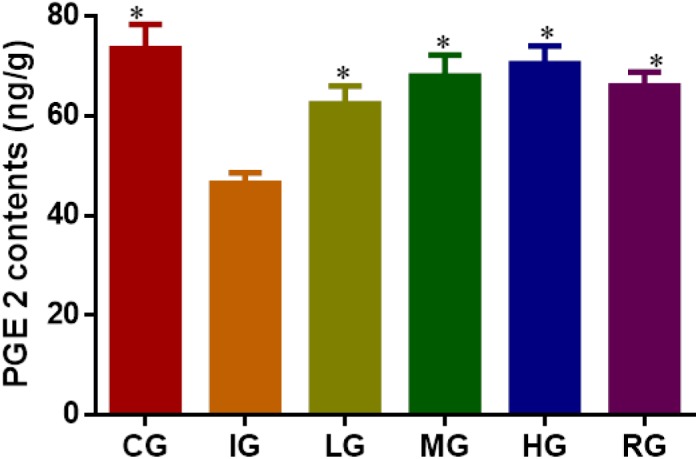
ELISA analysis for the effects of DNJ on the level of prostaglandin E2 (PEG2). Eight healthy mice were assigned as a CG and eight model mice were assigned as a model group (IG). The model mice received different concentrations of DNJ (10, 20, and 40 μg/kg daily) were assigned as LG, MG, and HG groups. The model mice were administrated with ranitidine (0.5 mg daily) as a positive control group (RG). *N* = 8 for each group. ^∗^*P* < 0.05 vs. the IG group.

The synthesis of mucosal PGE2 was markedly suppressed on the fourth day of ulceration induction in the mice (**Figure 10**, *P* < 0.05) and increased again by DNJ treatment with the increase of its dosage when compared to the mouse models (*P* < 0.05). The results suggest that DNJ treatment promotes PGE2 synthesis.

### DNJ Regulated Key Molecules Involved in NF-κB Signaling Pathway

**FIGURE 11:**
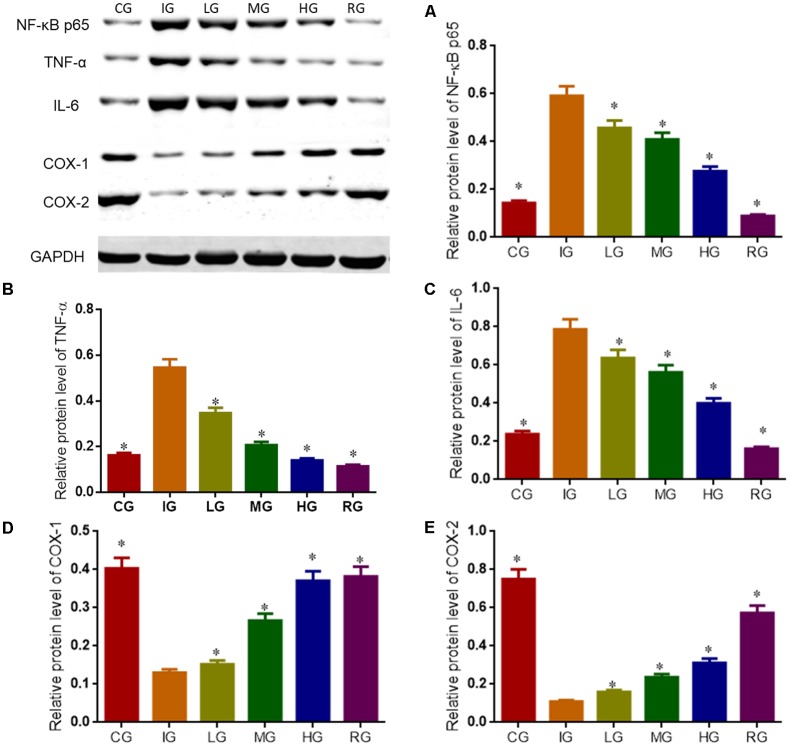
Western blot analysis for relative levels of main molecules in NF-κB signaling pathway. Eight healthy mice were assigned as a CG and eight model mice were assigned as a model group (IG). The model mice received different concentrations of DNJ (10, 20, and 40 μg/kg daily) were assigned as LG, MG, and HG groups. The model mice were administrated with ranitidine (0.5 mg daily) as a positive control group (RG). **(A)** Relative protein level of NF-κB p65. **(B)** Relative protein level of TNF-α. **(C)** Relative protein level of IL-6. **(D)** Relative protein level of COX-1. **(E)** Relative protein level of COX-2. *N* = 8 for each group. ^∗^*P* < 0.05 vs. the IG group.

Western Blot analysis showed that indomethacin treatment increased the levels of NF-κB p65, IL-6 and TNF-α, and reduced the levels of COX1 and COX2 when compared with CG (**Figure 11**, *P* < 0.05). Comparatively, DNJ inhibited inflammatory levels of GU mice by reducing the level of NF-κB p65, IL-6 and TNF-α, and increasing the levels of COX1 and COX2. Meanwhile, DNJ consumption prevented the activities of NF-κB signaling pathway by increasing PEG2 level (**Figure 10**), which is an inhibitor of NF-κB pathway.

## Discussion

The etiology and pathogenesis of GU remains widely unclear. Most of cases are believed to be related to bacteria (Cherkas et al., 2017; Sverden et al., 2017), immune diseases (Huang et al., 2000; Ayada et al., 2009), surroundings (Miles et al., 2012), and genes (Ha et al., 2017; Ma et al., 2017). Presently, the association between NF-κB and GU attracts some researchers (Akanda and Park, 2017; Arafa Keshk et al., 2017). NF-κB is one of chains of immunoglobulin subunit kappa, which enhances κB sequence-specific binding of NF. Some work demonstrates that continuous control of NF-κB will result in immunodeficiency and functional loss of peripheral lymphoid organs (Stoffel et al., 2004; Scuto et al., 2013).

The NF-κB pathway is often involved with inflammatory reaction and immune activities and has become an important target for many diseases. According to the information, we measured the expression of main molecules associated with NF-κB pathway in GU mice and compared with controls. Indomethacin treatment reduces PEG 2 level, and results in overexpression of inflammatory factors IL-6 and TNF-α. Meanwhile, high-level of the inflammatory factors will prolong pathological progress of GU.

**FIGURE 12:**
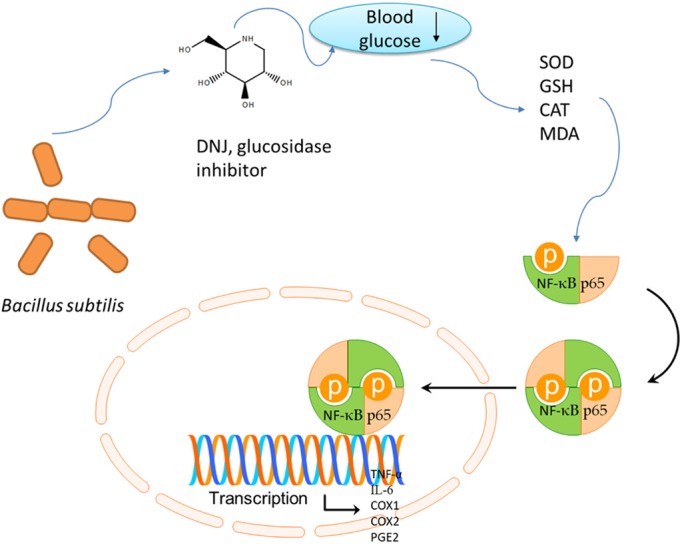
The cartoon figures to summarize the functional molecular mechanism of DNJ on gastric ulcer (GU) by affecting NF-κB pathway.

α-Glucosidase inhibitors have the potential for treatment of various diseases (Adisakwattana et al., 2004). Glucosidase inhibitor increase the glucose flow and inhibits hypoglycemic disorder by inhibiting oxidative stress (Suzuki et al., 2016). Nojirimycin consists of endocyclic nitrogen instead of the oxygen pyranosidic atom (**Figure 12**). The presence of a hydroxyl group at C-1 increase DNJ instability. DNJ is pH dependent and CH_2_OH group in DNJ plays an important role in promoting effective binding. The binding of DNJ (protonated inhibitor) to the glucosidase is driven by a favorable enthalpy. The additional ordering of bound solvent may reduce 1-deoxynojirimycin binding function with glucosidase (Zechel et al., 2003).

DNJ is an effective glucosidase inhibitor and can reduce blood glucose. The reduced blood glucose will increase antioxidant properties (Ribas et al., 2012; Adewale et al., 2016; Cai et al., 2017) (with increase of SOD, CAT and reduced GSH, and decrease of MDA) and anti-inflammatory (Shen et al., 2004) properties. Up-regulation of SOD, CAT and reduced GSH levels, and the down-regulation of MDA levels are associated with the inhibition of NF-κB pathway (An and Shang, 2018), which affect the expression of TNF-α, IL-6, COX-1, COX-2, and PGE2 (**Figure 12**). DNJ has been proven to improve lipid composition of GU models by reducing MDA level and cellular immune function by reducing the levels of IL-6 and TNF-α (**Figure 6**). Present results demonstrated that DNJ improved the activities of SOD, CAT and reduced GSH (**Figures 7A–C**), which would improve the antioxidant capabilities of GU mice. On the other hand, DNJ inactivated NF-κB signaling pathway, which was found to be associated with the expression of PGE2 (Hu et al., 2016; Tsai et al., 2017).

Inhibition of indomethacin on prostaglandin synthesis has been found to be associated with ROS formation, which is an important risk for GU pathogenesis (Bandyopadhyay et al., 2002; Bandyopadhyay and Chattopadhyay, 2006; Peng et al., 2008). It is necessary to understand the events to design new anti-ulcer drugs. Considering the side effects of most anti-GU drugs, exploiting natural products of probiotics maybe appropriate in GU therapy. *Bacillus subtilis* is a live microorganism that has many health-promoting effects (Ayala et al., 2017) and may produce potential drug for GU treatment. DNJ from *B. subtilis* increased SOD (**Figure 7A**), CAT (**Figure 7B**), reduced GSH (**Figure 7C**), and reduced MDA level (**Figure 7D**) of indomethacin-induced GU mice. Furthermore, inhibition of alpha-glucosidase may have antioxidant features by inhibiting the levels of 2,2-diphenyl-1- picrylhydrazyl, hydroxyl, and superoxide radicals, hydrogen peroxide and lipid peroxidation (Sahin Basak and Candan, 2013). DNJ is a potential glucosidase inhibitor and should have anti-oxidant properties.

Motilin, SP, SS, and VIP are closely associated with gastric functions. MOT and SP will be increased in GU development (Dudani et al., 2016). In conversely, SS (Coskun et al., 2013) and VIP (Kasacka et al., 2015) have protective functions for stomach. To understand the effects of DNJ on gastric functions, the effects of DNJ on MOT, SP, SS, and VIP in GU mouse models were measured via ELISA. The present findings demonstrate that DNJ can reduce the levels of MOT and SP, and increase the levels of SS and VIP (**Figure 8**). All these changes will be beneficial to control GU progression.

The remarkable increase in ulcer index and gastric volume following indomethacin-induced GU in mice, may attribute to ROS formation or prostaglandin decrease. Low-level prostaglandin level has been attributed to gastric injury and is associated with the etiology of GU, which is accordant with an earlier report (Hassan et al., 2016). PGE2 can regulate the activities of various cells, the production of inflammatory cytokines and have strong inhibition on cAMP-dependent modulation of NF-κB activity. Present findings demonstrated that DNJ played an anti-inflammatory role by increasing PGE2, COX1 and COX2 levels, and down-regulating NF-κB pathway, which was accordant with an earlier report (Min et al., 2002).

There are some limitations for the present work: (1) as a glucosidase inhibitor, exact functional molecule mechanism of DNJ remains unknown. (2) The present work only focuses on the association between different DNJ dosage and the changes of major proteins molecules in NF-κB signaling pathway. Gene interference and up-regulation may show more light on the mechanism. (3) The histology analysis and the levels related molecules were not measured on successive days. (4) The structural activity of DNJ was not explored in the present study, and more data with more molecules should be measured in the NF-κB signaling pathway. (5) The present work was still limited to animal models and clinical experiment was not performed. According to an earlier report, long-term consumption of DNJ (6 mg DNJ, t.i.d.) resulted in improved post-prandial glycemic control in the patients with impaired glucose metabolism (Asai et al., 2011). Further work is highly demanded to make sure the present conclusion and address these issues in the future.

## Conclusion

In sum, DNJ can effectively control GU development by improving antioxidant and anti-inflammatory capabilities of GU mouse models via NF-κB signaling pathway. The present findings are promising and support the use of DNJ as a source of natural anti-inflammatory and antioxidants. Overall, the attenuation of gastric affronts of indomethacin by administration of DNJ is indicative of its excellent gastric protective and antioxidant properties in GU mouse models. Efforts are ongoing to explore anti-GU activities of DNJ in *B. subtilis* and also harness their possible synergistic efficacy against GU.

## Author Contributions

XP and BL: conceived and designed the experiments, and wrote the paper. SL, XS, XL, HL, and TW: performed the experiments. LYG, LMG, SC, and YL: analyzed the data. BL: contributed reagents, materials, analysis tools.

## Conflict of Interest Statement

The authors declare that the research was conducted in the absence of any commercial or financial relationships that could be construed as a potential conflict of interest.
